# Ethnozoology among the Berbers: pre-Islamic practices survive in the Rif (northwestern Africa)

**DOI:** 10.1186/s13002-021-00466-9

**Published:** 2021-07-13

**Authors:** Aymane Budjaj, Guillermo Benítez, Juan Manuel Pleguezuelos

**Affiliations:** 1 Department of Zoology, Faculty of Sciences, University of Granada, 18071 Granada, Spain; 2 Department of Botany, Faculty of Pharmacy, University of Granada, 18071 Granada, Spain

## Abstract

**Background:**

Ethnozoological knowledge is less documented than ethnobotanical. With this field study, we aim to record and analyze the Riffian Berber knowledge about the use of animals in traditional human and veterinary medicine. Our research question is what is their knowledge of ethnozoological practices?

**Methods:**

We performed semi-structured interviews with local inhabitants in Riffian vernacular language. The reliability of the sampling effort was assessed by a rarefaction curve. Data were compared with previous studies in order to determine the geographical and historical extensions of described uses and possible conservation implications for the species used.

**Results:**

We obtained information regarding 107 ethnozoological uses based on 197 use reports. Among the 31 species used, mammals were most frequently cited. Diseases related to the traditional medicinal system were most frequently treated with these resources, as well as those of the respiratory, digestive, and musculoskeletal systems. Thirty percent of uses are associated with magico-religious practices. Only three of the species used are threatened at the global level, two of them extinct in the study area, indicating low potential damage to regional biodiversity from current practices utilizing native animals. Within modern Morocco, Riffians have continued practicing ethnozoological uses anathema to Islam, like the consumption of animals considered impure (dogs, jackals, wild boars, and hyenas).

**Conclusions:**

The use of primarily mammalian species and of many animal body parts is likely related to the Berber belief in homology between the area of the human body in which the ailment occurs and the corresponding animal body part. These findings unveil the nature of ethnozoological practices, highly linked to folklore and culture-bound conditions, and lacking in the Western empirical rationale for nearly one third of reported uses. The consumption of animals considered impure according to Islam was probably initiated before the conquering of the Maghreb by Arabs in the seventh century and was maintained through the secular isolation of Riffians in mountain areas. This can reflect traditional healing habits being maintained over thousands of years.

**Supplementary Information:**

The online version contains supplementary material available at 10.1186/s13002-021-00466-9.

## Background

Ethnozoology is the study of people and culture in relation to surrounding animals [1]. Mason [2], who proposed the term, stated that all fauna encountered in a region enters directly or indirectly into the thoughts and lifestyles of each human group. Today, it is considered an interdisciplinary field of research [3] that examines historic, economic, anthropologic, and environmental aspects of human and animal relationships [1]. The discipline is currently well integrated into current anthropological theoretical discussions [4], such as how humans obtain and transmit knowledge, use their available resources, and conserve both the resource and its associated knowledge. Moreover, ethnozoological studies can also play an important role in biodiversity conservation, as it is difficult to design effective wildlife conservation and management strategies in some regions without considering ethnozoology [5].

Ethnozoology in Africa has performed, and continues to perform, a fundamental role within numerous ethnicities. For example, in Egypt, Khushmaan Ma’aza Bedouins, due to the diversity of reptiles in desert environments, often use them for medicine [6]. The use of animals for human health is also still very accepted in Nigeria [7] and Central Sudan [8], and a large proportion of people in South Africa will visit a traditional healer at least once in their lifetime [9].

In Morocco, traditional medicine is locally preserved because it is deeply rooted in the knowledge of the rural culture [10] and because of the distance from the nearest health centers in rural areas [11]. Fifty-five percent of the population was living in rural zones, under poor-quality sanitary conditions while frequently using traditional medicine (mainly plant-based, followed by animal-based) at the end of the twentieth century [12]. The current depopulation of rural areas, along with an aging population [13], makes us think that within a few generations, ethnozoological knowledge will be reduced and, in some cases, disappear. While ethnobotanical studies in Morocco are relatively numerous (e.g., [14, 15]), that is not the case for ethnozoology. Morocco’s proximity to Europe and the early presence of European embassies in the city of Tangier instigated studies of Moroccan ethnology (including ethnozoological information) as early as the first half of the nineteenth century [16–23]. After the country’s independence (in 1956), however, no further information was published on the subject until the beginning of the twenty-first century. This mostly included studies focused on the impact of ethnozoology on wildlife conservation and geographically centered on the Atlantic plains and southern Morocco [10, 24–28].

The present field-study is based in the mountainous, highly biodiverse (though less prospected by naturalists) region of the Eastern Rif in Northern Morocco. During the Spanish protectorate of Morocco (1912–1956), this region began to be visited by herpetologists [29], mammalogists [22], and ornithologists [30]. Its herpetofauna has recently been inventoried [31, 32], although the fauna of other continental vertebrate groups (fishes, birds, and mammals) is still poorly known [33–35]. The Rif region is characterized by its indigenous inhabitants, the Berbers, the native ethnic group in the region. The Berbers are comprised of sedentary farmers and have been present long before Islamization in the Middle Ages. Morocco is currently the Maghrebian country with the highest Berber population [36] and houses the purest groups of this ethnicity [37]. Due to their bellicose character, they have rarely been subdued by other peoples [21]; during the seventh century Arabic conquest, they were Islamized but not Arabized [20]. The region where they thrived was considered *Bled as-Siba* (lawless area out of the control of the Moroccan Sultans) until 100 years ago [38], and in the Berber language, they proclaim themselves *Imazighen* (free men and women). They constitute two million inhabitants, and their language (Amazigh) and writing (Tifinagh) are of clear pre-Islamic origin [36]. Despite a notable genetic admixture with other Northwestern African populations [39], their cultural differences and independent character, particularly among Riffians, bring added interest to the ethnozoology of this region in regard to the exploration of the maintenance of pre-Islamic habits. In North Africa, the Berber religion was based on Phoenician and Punic deities, with a god (Baal) and a goddess (Tanit), and lacks prohibitions established by Islam [40]. This paganism (with respect to Islam) was not completely eliminated; Berbers of the Rif adopted the Islamic religion, but it seems they did not abandon some pre-Islamic customs, and continue to practice them in a hidden way [21]. We should also consider that the cost of modern medicine has been beyond the reach of the Moroccan Berbers until few years ago [11], and the study area currently maintains rather scant and scattered medical facilities (authors, per. obs.). Previous anthropological studies on the Moroccan medicinal system (as part of Arabic medicine) pointed to a pluralistic and ambiguous system from two medical traditions: one indigenous and magico-religious, the other alien, scientific, and secular [41, 42], reflecting a partial syncretism between Prophetic medicine (established after the Prophet’s death), and the Galenic humoral medicine (introduced by Arabs [41]). Indeed, in Morocco, religion and beliefs play an important role in medical diagnosis and treatments, and demons (*jnun*) are central in explaining daily events of reality [43].

Within this socioeconomic context of the territory, and due to the fact that several studies have pointed to a wide ethnobotanical knowledge among Berber people [44, 45], we focused our study on the traditional use of fauna in the Rif mountains. This study records the species with ethnozoological interest in the region, the parts of the animal used, the modes of application, the treated diseases, the permanence of this knowledge, the sociodemographic profile of the interviewees, and the impact of these practices on regional biodiversity [46]. Our research questions were what knowledge do the Riffian Berbers have about ethnozoological practices and what could the origin of these practices be? As a secondary aim, we also raised the question of whether these practices have any implications for the conservation of the species. Our objectives were (1) to fill the gap in the knowledge of the use of animals in traditional human and veterinary medicine, or magic/sorcery, in northern Morocco, a region where a traditional pharmacopeia continues to provide remedies for use in healthcare by Riffian Berbers [12]; (2) to analyze the ethnozoological uses under historical and ethnopharmacological points of view in order to understand the origin of this knowledge and if some uses are medically beneficial or rather can be better associated with ritual traditions or religious faith; and (3) to analyze the animals utilized with respect to possible implications for their conservation.

## Methods

### Study area

**Fig. 1:**
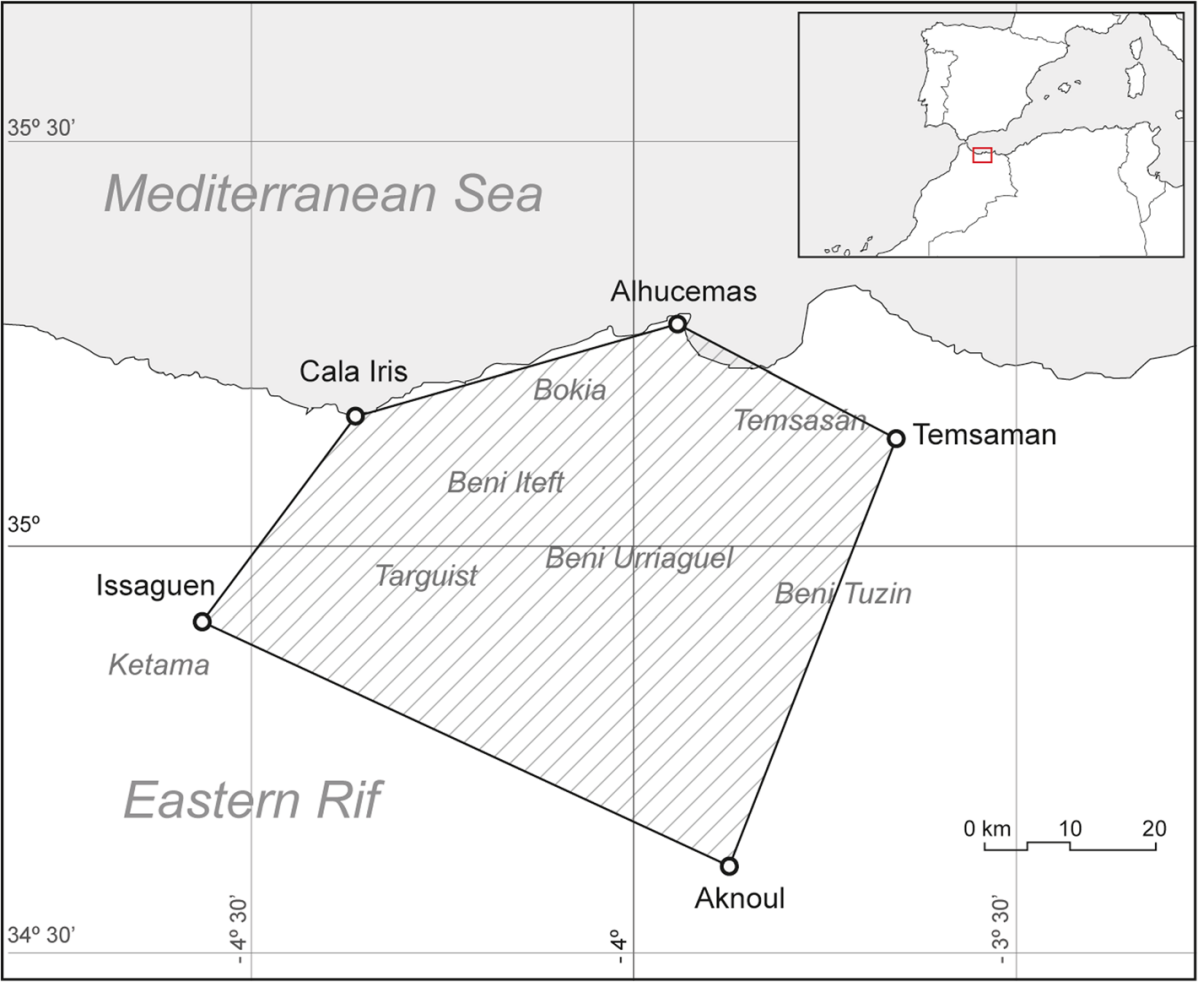
Study area in Northern Morocco and location within the Western Mediterranean. Name and approximate range of included Berber tribes in italics

Located within the northern Moroccan province of Tangier-Tetuan-Al Hoceima and bounded by Al Hoceima (35.2515^o^, −3.9340 ^o^), Cala Iris (35.1404 ^o^, −4.3770 ^o^), Issaguen (34.9165 ^o^, −4.5685 ^o^), Aknoul (34.6516 ^o^, −3.8661 ^o^), and Temsaman (35.1280 ^o^, −3.7321 ^o^), the study area covers 3500 km^2^. As part of the Rif mountains, the territory is fundamentally mountainous (0–2000 meters above sea level) and has a Mediterranean climate. Our study includes the Berber tribes of Beni Waryager, Bokoya, Beni Tuzin, Targuist, Temsaman, Ketama, and Beni Iteft (Fig. 1).

### Survey methods

In order to gather ethnozoological information in the study area, we performed semi-structured interviews on randomly selected local inhabitants in the field. With their aid, the snowball method was used to locate other informants. While gathering data, we followed the ethical standards set by the International Society of Ethnobiology and the consensus statement on ethnopharmacological field studies [47] as a rule. Interviews were performed in situ between December 2017 and April 2019 following the standard methodology used for ethnobiological data collection [48, 49]. Semi-structured oral surveys were chosen, allowing interviewees the freedom to respond and further comment on what they think is important. We initially explained the purpose of our questions and obtained consent from the interviewee to become a participant in our study. The language used was mainly Riffian, the first language of the respondents, although we also used Moroccan Arabic dialect (Darija) according to the preferences of the respondents.

Our questions were concerned with identifying which animal species in the region were involved in any medicinal, magico-religious, or folkloric use, or have to do with any other associated cultural information. For all uses, we also gathered data on the parts of the animals used, preparation methods and modes of application of the remedies, the disease(s) or symptom(s) treated, and determined to what extent these practices are still maintained. In an extension of our study into biodiversity conservation, we also inquired as to whether the animals were hunted locally or traded in local markets (*souks*), and if any population decrease in density (= numbers) was observed (see also [47, 50, 51]). With these last questions we aimed to estimate the impact of ethnozoological practices on the regional biodiversity, according to the conservation status (IUCN Red List categories) of the species used by locals. An anthropological extension of our study included anonymous socio-demographic information, as we also gathered data on our informants (age, gender, job, locality, and tribe).

During the interviews, animals were mentioned using vernacular names. For the association of vernacular names with their scientific counterparts, scientific names were directly interpreted from unambiguous local names in most cases (e.g., ærʁəm, camel). In others, this was determined from descriptions provided by the interviewees (e.g., “lizard with reddish spot in the throat area” corresponds unequivocally to *Psammodromus algirus*)*.* In some cases, we also showed the interviewees field guides covering the region [35, 52, 53], which helped to identify the species. Additionally, we obtained photographs of some of the animal species here considered, used to refine the identification. Lastly, it is worth mentioning that the authors have extensive experience with continental vertebrates in the region (e.g., [54–56]). In all cases, the identification was as conservative as possible, and in few cases, we could only reach the family or group (see the “Results” section).

### Data analysis

Species vernacular names in both Riffian and Moroccan Arabic were collected and recorded. For the first time in an ethnozoological study, Riffian names have been written in Riffian format (Tifinagh, in the script for this language accepted by the Royal Institute of Amazigh Culture [IRCAM] [57];). Westermarck [58] previously presented animal names in the Tifinagh of some tribes, but only as a phonetic transcription. Our phonetic transcription from Riffian to English follows the rules of the International Phonetic Alphabet (IPA). We also consulted the Arabic names previously published [24, 31].

Our results show the primary data obtained, presented in the form of descriptions of uses (animals, part of the animal, preparation, and modes of administration) and frequency-of-use reports as a recommended standard [47, 59]. As we are aware that there are difficulties in establishing the disease etiology or correlating them with a condition in Western medicinal systems [60], we asked directly for the local use, without asking about how conditions are diagnosed or the possible causes according to the local medical system. Conditions and symptoms were recorded according to their emic descriptions as described by our informants, and later organized into pathological groups for analysis following the WHO International Classification of Diseases (ICD-11, version 04/2019 [61]; Table S1). Despite its limitations for ethnomedicine [62], we used ICD-11 because it is the standard diagnostic tool for epidemiology, health management, and clinical purposes, and the association of conditions with pathological groups is accurate and intuitive. For animal parts, modes of application, names of diseases, and maintenance of the ethnozoological uses, our records follow the terminology presented in [16, 50]:

- Animal parts: blood, bones, ears, entire, tusks, feces, feathers, fat, gut, honey, horns, hooves, jaw, liver, meat, milk/colostrum, poison, skin, spines, unknown, urine.
- Application modes: amulet, contact with smoke when burned, drinking, ingestion, ingestion of ashes, ingestion of oil, ingestion of poison, keeping at home, rubbing, sleeping in leather, smoke inhalation when burned, steam inhalation, sting on the affected area or on the body, topical, wash-over.
- Origin of the animals: hunted or bought in stores and souks.
- Current maintenance of the ethnozoological uses: affirmative or negative.
- Population tendency of wild animals used: stable or decreasing.

### Literature review

In order to gather previous references regarding the uses described by our informants, we performed a literature review of some of the most important anthropological sources in the area (e.g., [43, 63, 64]). We also consulted two important historical sources for natural medicinal products: Dioscorides’ *Materia Medica* and the personal comments of his Spanish translator, Laguna [65], and the *Compendium of simple medicaments* of Ibn al-Baytar [58], whose importance for the ethnobiology of Morocco was previously highlighted [64, 66].

For comparing the proportion of species used vs. the total wild species living in the area within each group of animals, we compiled a catalogue of the wild species of amphibians, reptiles, birds, and mammals extant or recently extinct in the Eastern Rif (Table S2). Literature sources for this catalogue include [32] for amphibians and reptiles, [34] for birds, and [35] for mammals. Recent genetic studies [67] suggest only one jackal-like canid (the African wolf, *Canis lupaster*) be included in our study area.

### Statistical analysis

For continuous variables (e.g., the age of the interviewees), we provided the mean, standard deviation, and sample size. For discrete variables, we provided the same metrics and frequency distributions, and comparisons were performed using contingency tables and chi^2^ statistics (e.g., the distribution of species by animal group used by ethnozoology and the distribution found in the wild). The relationship between discrete variables was assessed using the Spearman’s rank correlation coefficient (the relationship between the age of the interviewees and the number of species reported). Reliability of the sampling effort was assessed using a rarefaction curve, by adjusting the relationship between the number of surveys carried out and the accumulated species richness to a logarithmic distribution [68, 69]. Alpha was set at 0.05. These calculations have been made with the STATISTICA Statsoft v10 program.

## Results and discussion

Twenty-nine vertebrates and two invertebrates (91.2% at the species level) were reported as being utilized for ethnozoological purposes. The species and their uses, sorted by the main animal group and scientific name, can be seen in Table 1, together with the common English and Arabic names, the Riffian name with the phonetic transcription into English, the tribes where the uses were recorded, the use, part of the animal used, mode of administration, and citation number for the animal and condition (use reports). A total of 107 uses are described in Table 1, with 197 use reports.

**Table 1:** Animal species with ethnozoological uses reported by Riffian Berbers of the Eastern Rif (N Morocco; name of the tribes included). Species are arranged in alphabetical order within taxonomic groups for the readers’ convenience. In the use column, numbers between brackets follow the ICD-11 ([62], Table S1). D = Dioscorides, IB = Ibn al-Baytar (coincident uses), M = Magico-religious use, V = Veterinary use, * = Use not currently practiced by at least one informant, UR = Use reports

Scientific name (family)	Common name	Riffian name [phonetic transcription into English]	Arab name	Tribe	Use	Body part used	Application mode	UR	Total UR
Insecta									
*Apis mellifera* (Apidae)	Bee			Beni Waryager Bokoya Targuist	Stomach pain (13)	Honey	Ingestion	1	4
					Against the cold (23) D IB	Entire	Sting in affected area or in the body	2	
					Reumatism (15)^a^	Entire	Sting in affected area or in the body.	1	
Lampyridae	Firefly			Beni Tuzin	Antivenom (22)	Entire	Ingestion (dry, pulverized).	2	2
Amphibia									
*Sclerophrys mauritanica* (Bufonidae)	Moroccan toad			Ketama	Rough sole (14) M^a^ D IB	Entire	Topical	1	1
Reptilia									
*Chamaeleo chamaeleon* (Chamaeleonidae)	Common chameleon			Targuist Beni Waryager	Stomach pain, diarrhea (13)	Blood	Drink	1	3
					Calmative (21)^a^	Blood	Drink	1	
					Against evil eye (26) M^a^	Entire	Inhale smoke when burning	1	
*Psammodromus algirus* (Lacertidae)	Large psammodromus			Temsaman	Reumathism (15) M^a^	Entire	Amulet	1	1
Ophidia	snake			Bokoya Beni Waryager	Cancer (2)^a^	Meat	Stewed	4	7
					To kill someone (22)^a^	Venom	Venom Ingestion	2	
					Rough skin (14)^a^	skin	Rubbing	1	
*Testudo graeca* (Testudinidae)	Mediterranean spur-thighed tortoise			Bokoya, Beni Waryager Temsaman Targuist Beni Tuzin	Lactation cessation (5)^a^	Meat	Stewed	4	22
						Entire	Ashes ingestion	6	
					General healing (26)^a^	Meat	Stewed	3	
						Entire	Ashes ingestion	1	
					Stomach pain (13)^a^	Meat	Stewed	1	
					Hypothermia (23)^a^	Meat	Stewed	1	
					Leucodermia (14)^a^	Meat	Mixed with henna	1	
					Start walking delay (20) M^a^	Entire	Wash over	1	
					Against evil eye (26) M^a^	Entire	Wash over	1	
					Reumathism (15)^a^	Entire	Wash over	1	
					Pneumonia, bronchitis (12) M	Entire	Keep in courtyard	2	
Aves									
*Athene noctua* (Strigidae)	Litle owl			Beni Waryager	Sorcery (bad omen) (26) M	Entire		1	1
*Alectoris barbara* (Phasianidae)	Moroccan partridge			Beni Waryager	Measles (1)	Entire	Stewed	2	2
*Bubulcus ibis* (Ardeidae)	Cattle egret			Beni Waryager	General healing (26)^a^	Entire	Stewed	1	1
*Columba livia* (Columbinae)	Rock pigeon			Bokoya Beni Waryager	General healing (26)^a^	Entire	Stewed	2	3
					General stimulant (21)^a^	Entire	Stewed	1	
*Corvus corax* (Corvidae)	Raven			Targuist Bokoya	Generate insomnia (7) M^a^	Entire	Stewed	2	4
					Improve sight (9)^a^	Entire	Stewed	1	
					Asthma (12)^a^	Blood	Drink	1	
*Gallus gallus* (Phasianidae)	Hen			Beni Waryager Bokoya	General healing (26) D IB	Meat	Stewed	1	3
					Be cold (23)	Entire	Stewed	2	
*Upupa epops* (Upupidae)	Hoopoe			Beni Waryager Targuist Beni Tuzin	General healing (26)^a^	Blood	Drink	3	13
						Meat	Stewed	2	
					Lack of concentration (6) M^a^ IB	Blood	Drink	2	
					Generate insomnia (7) M^a^	Blood	Drink	1	
						Meat	Stewed	1	
					Against bad luck (26) M^a^	Bone	Mixed with henna	1	
					Sorcery (26) M^a^ IB	Entire	No information	1	
						Bone	No information	1	
						Feather	No information	1	
Mammalia									
*Atelerix algirus* (Erinaceidae)	Algerian hedgehog			Beni Waryager Targuist Temsamán Bokoya Beni Tuzin	Against evil eye (26) M^a^	Spines	Smoke in contact with eyes when burning	2	18
					Weakness, tiredness (21)	Meat	Stewed	1	
					General healing (26)^a^	Meat	Stewed	2	
						Blood	Drink	3	
					Stomach disorder, diarrhea (13)^a^	Blood	Drink	2	
						Entire	Ashes ingestion	4	
					Asthma (12)^a^	Blood	Drink	1	
					Hypothermia (23)^a^	Entire	Ashes ingestion	2	
					Livestock general healing V^a^	Gut	Inhale the smoke when burning	1	
*Bos taurus* (Bovidae)	Cow			Bokoya	External hemorrhage (23)^a^	Fat	Topical	1	4
					Hyperglycemia (5)^a^	Fat	Topical	1	
					Bronchitis, pneumonia (12)^a^	Meat	Stewed	1	
					Renal disturb (16)^a^	Meat	Stewed	1	
*Bos taurus* (Bovidae)	Bull			Bokoya	Snake repellent (26) M^a^	Horn	Inhale vapor when boiled	1	4
					Hair strengthening (14)^a^	Urine	Rubbing	1	
					Vomit (13)^a^	Liver	Drink	2	
*Camelus dromedarius* (Camelidae)	Camel			Temsamán Beni Waryager Bokoya	Fever (21)	Meat	Stewed	1	26
					Join pain (15)^a^	Meat	Stewed	1	
						Fat	Topical	1	
					Aging (21)	Meat	Stewed	1	
					Painful urination (16)	Meat	Stewed	1	
					General healing (26)	Meat	Stewed	1	
						Meat	Topical	1	
						Urine	Topical	1	
						Milk	As a drink	1	
					Herpes (1)	Meat	Topical	1	
					Sight improving (9) M	Meat	Boiled. Eyes in contact with steam	1	
					Hemorrhois (20)	Fat	Topical	1	
					Diabetes (7)	Fat	Topical	4	
						Milk	Drink	4	
					Snake repellent (26)	Fat	Topical	1	
					Lices (1)	Urine	Rubbing	1	
					Dandruff (20)	Urine	Rubbing	1	
					Hair color loss (14)	Urine	Rubbing	1	
					Nose mucus excess (12)	Urine	Topical	1	
					Nose-bleed (12)	Urine	Topical	1	
*Canis lupaster* (Canidae)	African wolf			Bokoya Beni Waryager Targuist Temsaman	Against fear (26) M^a^	Meat	Stewed	1	10
					General healing (26)	Meat	Stewed	1	
						Entire	Ashes ingestion	1	
					Stay awake (7) M^a^	Meat	Stewed	1	
					General weakness (21) M	Meat	Stewed	1	
					Baldness prevention (14) M^a^	Fat	Topical	1	
					Against coldness (23)^a^	Fat	Topical	1	
						Entire	Ashes ingestion	2	
					Stomach pain (13)^a^	Gut	Oil ingestion after frying	1	
*Canis lupus familiaris* v(Canidae)	Dog			Targuist Temsamán	Anti-envenomation (22) M^a^	Blood	Drink	1	2
					Stomach cancer (2) M^a^	Meat	Stewed	1	
*Capra aegagrus* (Bovidae)	Domestic goat			Bokoya Beni Waryager	Anti-envenomation (22) M^a^	Meat	Stewed	1	3
					Recovery after fall (15) M^a^	Skin	Sleep on the leather	1	
					Against fear (26) M^a^	Dung	Heated by charcoal above the patient	1	
*Equus asinus* (Equidae)	Donkey			Bokoya Targuist Beni Waryager	Lactation cessation (5)^a^	Milk	Drink	3	9
					Make a person acquire an idiot behavior (26) M^a^	Ear	Stewed	6	
*Equus caballus* (Equidae)	Horse			Beni Waryager	Migraine (8)^a^	Hoof	Inhale smoke when burning	1	1
*Felis lybica* (Felidae)	Domestic cat			Beni Waryager	Nail troubles (20) M^a^	Ear	Nails inside cat ears	4	4
*Gazella cuvieri* (Bovidae)	Cuvier’s gazelle			Beni Waryager	Diuretic (16)^a^	Meat	Stewed	1	1
*Hyaena hyaena* (Hyaenidae)	Striped hyena			Beni Tuzin	Sorcery (26) M^a^	Unknown	No information	2	2
*Mustela nivalis* (Mustelidae)	Weasel			Beni Tuzin	Sorcery (26) M^a^	Entire	No information	1	3
					Asthma (12)^a^	Blood	Drink	1	
					General healing (26)	Meat	Stewed	1	
*Oryctolagus cuniculus* (Leporidae)	Rabbit			Bokoya	Epilepsy (8) M^a^	Meat	Stewed	1	3
					Lower body paralysis (8) M^a^	Meat	Stewed	1	
					Urinary retention (16)^a^	Meat	Stewed	1	
*Ovis aries* (Bovidae)	Sheep			Beni Waryager Bokoya	Cough (12)^a^	Meat	Stewed	1	4
					Chest pain (12)^a^	Meat	Stewed	1	
					Painful urination (16)^a^	Meat	Stewed	1	
					Against fear (26) M^a^	Dung	Heated by charcoal above the patient	1	
*Ovis aries* (Bovidae)	Lamb			Beni Waryager Bokoya	Oral inflammation, dental problems (20)	Jaw	Heated and topical	2	2
*Panthera leo* (Felidae)	Lion			Beni Waryager	Back pain (15)^a^	Fat	Heated and topical	1	3
					Reumathism (15)^a^	Fat	Heated and topical	1	
					Heart weakness (11)^a^	Meat	Stewed	1	
*Sus scrofa* (Suidae)	Wild boar			Bokoya and Beni Waryager	Stomach pain (13)^a^	Gut	Stewed	1	5
					Gut troubles (13)^a^	Gut	Stewed	1	
					Against evil eye (26) M^a^	Tusks	Amulet	3	
*Vulpes vulpes* (Canidae)	Fox			Beni Waryager Bokoya Targuist Ketama Temsamán Beni Tuzin	Join pain (15)^a^ IB	Meat	Stewed	1	26
					Asthma (12)^a^	Meat	Stewed	1	
						Entire	Ashes ingestion	6	
						Blood	Drink	6	
					Livestock general healing V^a^	Meat	Stewed	1	
					Children weakness (21)^a^	Entire	Ashes ingestion	1	
					General healing (26)^a^	Entire	Ashes ingestion	7	
					Tuberculosis (12)^a^	Entire	Ashes ingestion	1	
					Cancer (2)^a^	Blood	Drink	2	

Species are arranged in alphabetical order within taxonomic groups for the readers’ convenience. In the use column, numbers between brackets follow the ICD-11 ([62], Table S1)

*D* Dioscorides, *IB* Ibn al-Baytar (coincident uses), *M* Magico-religious use, *V* Veterinary use, *UR* Use reports

^a^Use not currently practiced by at least one informant

**Fig. 2:**
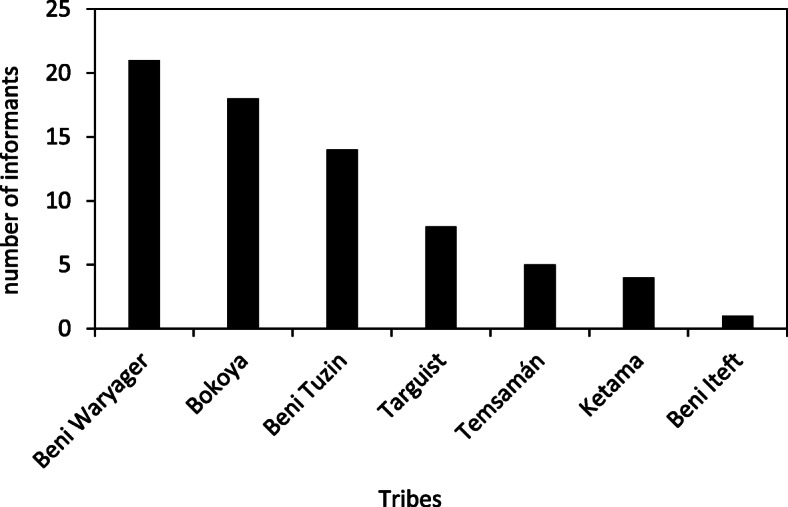
Distribution of informants per ethnic tribe

**Fig. 3:**
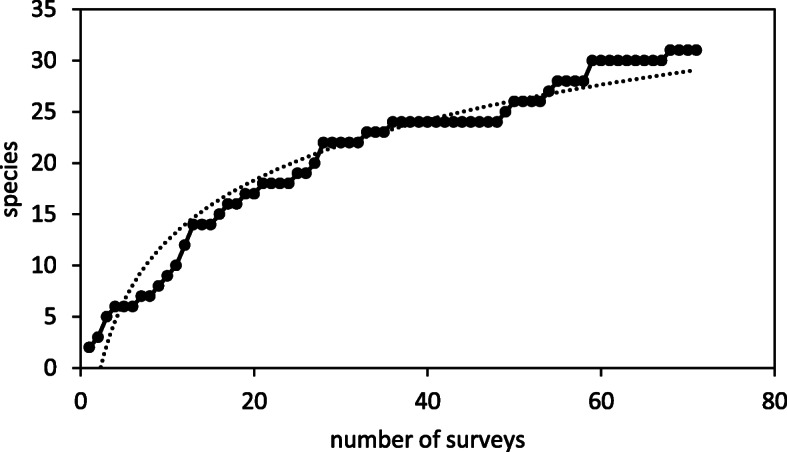
Accumulation curve. Number of species with ethnozoological use in relation to the number of surveys (interviews). y = 8.4811ln(x) – 7.092, *r*^2^ = 0. 9396, *p* < 0.000

We obtained 71 valid semi-structured surveys, with most interviewees representing the tribes of Beni Waryager, Bokoya, and Beni Tuzin (Fig. 2). We considered this interview effort to be enough, since the relationship between surveys (data collection) and species with ethnozoological uses (cumulative number of species cited in the interviews) reached an asymptotic curve with the horizontal (Fig. 3; see [48]). Therefore, to achieve a 5% increase in accumulated richness (i.e., 32.5 instead of 31 species), it would be necessary to gather 107.1 interviews (36 additional interviews, more than 50% of the current sample size). Moreover, the number of species with ethnozoological use found here exceeds the value for the same variable in other ethnozoological studies in the Mediterranean region [16, 70, 71], although it is inferior to the number obtained in tropical regions [72]. Thus, we consider our sample size suitable to depict the number of animal species used for ethnozoology in the study area. It would be more beneficial to survey new mountain systems than to increase the sample size in the same region. Except for *Upupa epops* (probably due to religious influence as this species is often quoted in the Muslim holy book), animal names obtained during our study are first represented with their written equivalents in Tifinagh.

### Sociodemographic profile of interviewees

Interviewees were mainly men (50 men vs. 21 women), likely due to our field methodology [73]. There were no significant differences between women or men regarding the mean number of animals reported as being used in ethnozoology (women, 2.6 ± 1.8, *n* = 21; men, 1.9 ± 1.2, *n* = 50, Mann-Whitney U test, U = 372.0, *p* = 0.07), nor between the total number of uses reported (women, 3.2 ± 2.0, *n* = 21; men, 2.5 ± 1.7, *n* = 50, Mann-Whitney U test, U = 395.5, *p* = 0.14). These data are different from those observed in ethnobiological studies on Berber nomadic shepherds, where plant use for veterinary purposes is mostly a male-dominated practice [74]. The mean age of interviewees was 53.0 ± 14.4 years (range 21–80, *n* = 71). Although there is a tendency to interview the elderly in ethnobiological studies ([49, 74]) and Riffian informants confirmed the elderly were the best repositories for oral tradition, we did not find significant relationships between the age of interviewees and the number of species (r_s_ = 0.082, *n* = 71, *p* > 0,05) or the number of uses (r_s_ = −0.030, *n* = 71, *p* > 0.05) reported.

### Animals with ethnozoological uses

**Fig. 4:**
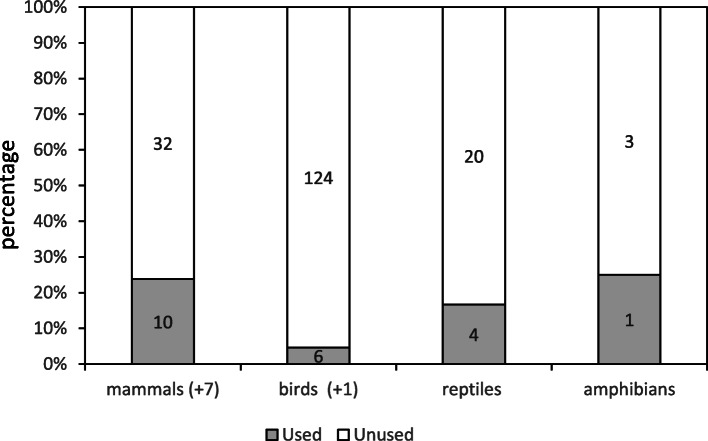
Percentage of used and unused wild animals in the region per animal group (from data in Table 1 and Table S2; no species number data available for Insecta). Species number in brackets for each category, and domestic species per animal group between parenthesis

Vertebrates with ethnozoological uses comprise 14% of 200 wild species living in the study area (Table S2), plus nine domestic species (eight mammals: bull/cow, camel, cat, dog, goat, donkey, horse, sheep/lamb, and a bird: hen). Thus, most vertebrates utilized are wild in the territory (69%, 20/29, see Table 1). As observed in other regions (e.g., [16, 51]), mammals are first in the number of species utilized for ethnozoological purposes (17 species and 34.7% of the mammals living in the study area, Table S2), followed by birds (7 species, 5.4%), then reptiles (4 species, 16.6%), and lastly amphibians (1 species, 25%; Fig. 4). Culturally, Berbers consider reptiles earthly forms of the devil [43, 63] and, perhaps for this reason, they also appear with rather high species frequency in surveys. Nijman and Bergin [10] found up to nine reptile species traded in the medinas of western Morocco, and intended for use in medical and/or magico-religious procedures. There is a statistically significant difference between the distributions of the number of species within animal groups used in ethnozoology and the number of species within those same groups living in the region (amphibians and reptiles are considered a single group here because of statistical constrains; 2 x 3 contingency table, chi-square = 148.49, *p* = 0.001). This is mainly precipitated by the low percentage of birds having ethnozoological uses coupled with the high bird species diversity in the study area (underused taxonomical group; Fig. 4; Table S2).

### Description of ethnozoological uses

Most of the described uses (105; 98%) aim to treat or heal human conditions, although 2 of them are veterinary (2%). Among Berber pastoralists across the Rif mountains, the ethnoveterinary uses (mostly botanical), however, are much more important [74]. For each animal species utilized in the Eastern Rif (Table 1), we discuss the results in a geographical (mostly within Morocco) and historical context.

#### Insecta

*Apis mellifera* Linnaeus, 1758: honey is one of the most appreciated and valued natural products introduced to humankind, and has, among others, demonstrated anti-inflammatory properties [75]. Bee venom is also used in some territories as a Complementary Therapy (apitherapy) to treat rheumatism [76]. It was also utilized for magical and aphrodisiac purposes in the Fez region [43]. The value of honey for treating symptoms of the common cold was mentioned by both Dioscorides and al-Baytar [67, 68].

*Lampyridae* Latreille, 1817: Hunted at night, it is dried and crushed to make a pâté that is ingested to treat poisoning. The species is probably *Pelania mauritanica* (Linnaeus, 1767), and its use has not been previously cited in Morocco.

#### Amphibia

**Fig. 5:**
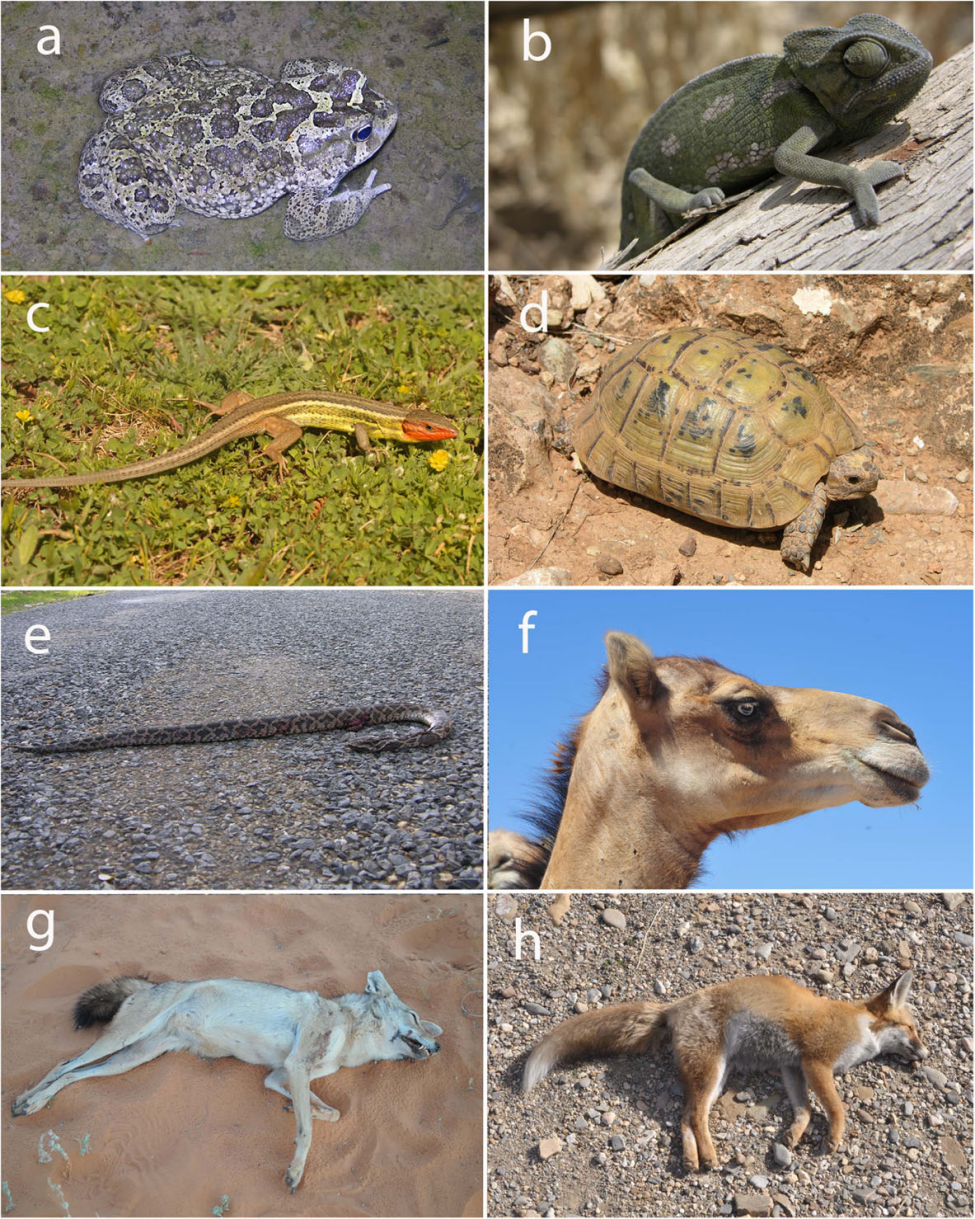
Photographs from the study area and surrounding regions of some included animal species. Photographs by J.M. Pleguezuelos

*Sclerophrys mauritanica* (Schlegel, 1841) (Fig. 5a): The body of a living Moroccan toad is longitudinally split by a knife, and the patient’s foot is inserted into it to soften the sole (as an emollient). Berbers from the Atlas opened a frog, applied it to the body of the patient, and left it there for a day or a night as a cure for abscesses [43]. This use was previously described by Dioscorides for fried frogs [65] and by Al-Razi (Rhazes, ca. 865 - ca. 925; cited by Ibn al-Baytar [58]) as a strong emollient. Among the Beni Waryager, there is a belief that toads possess extraordinary powers; if they are killed, the killer will suffer fever as punishment and if you smile or show it your teeth, they will fall out [43]. In Southern Morocco, this toad is dried and powdered to make toxic potions [24].

#### Reptilia

*Chamaeleo chamaeleon* (Linnaeus, 1758) (Fig. 5b): The chameleon is believed to possess magical powers [23, 43, 77] (e.g., sorcery uses related to the fidelity of couples). Moroccans believe that it is venomous, with a deadly bite and herbivorous diet [77], and that the chameleon is a man turned into an animal as a punishment for betraying the prophet by revealing his hiding place [24]. Chameleons split open alive were a remedy for wounds [17], and inhaling the smoke produced when burned is a remedy for sickness [10]. Sahrawi people in Western Sahara use its skin to treat boils, wounds, toothaches, and as anti-venom, and its eggs are used as an antidote for snakebites, to treat boils and skin abscesses [25]. In Dioscorides’ time it was believed that chameleon’s blood depilated the eyelashes [65], and Al-Baytar also cited this use [58]. This reptile species is frequently observed being traded in medinas in western Morocco for medicinal and sorcery purposes [10].

*Psammodromus algirus* (Linnaeus, 1758) (Fig. 5c): An individual must be put into a cylindrical container and then into the pocket; rheumatism will disappear when the animal has died. Temsaman women burned it underneath them and then inhaled the smoke to treat sterility [43]. In other areas of the Western Mediterranean, this species and other lizards are cited for musculoskeletal problems, like rheumatism [16, 24].

Snakes: Interviewees differentiated between venomous and non-venomous ophidians and cited few uses for non-venomous snakes (probably *Hemorrhois hippocrepis* (Linnaeus, 1758)). The use of ophidian venom must correspond to *Daboia mauritanica* Gray, 1849 (Fig. 5e), the only viperid species coinciding with the elevational range of our study area [31]. Some authors [77] point out that Moroccans do not generally distinguish venomous from non-venomous snakes, possibly explaining why snakes have not been identified to species level in this study. Other Moroccan natives are well aware of venomous ophidians, however [56]. In the literature, we find many additional uses for these reptiles. For instance, in Beni Tuzin, due to the excellent eyes of these animals, the shed skin was rubbed over the eyes to cure excessive tearing [23, 43]. Shed snake skin is also used to treat repeated eruptions of furuncles (furunculosis), and when liquefied, it can be used to calm ear infections, vitiligo, and alopecia [24]. Shed snake skin, mixed with salt, alharma (*Peganum harmala* L.), and alum, is burned in rituals to acquire the condition of healer [23, 24]. In the ancient literature, serpent’s skin was used for several conditions, including ear, tooth, and eye problems, warts, and hemorrhoids [58, 65]. The use of ophidian venom to kill other people for revenge or love problems is common in Morocco; venom is mixed with couscous, tea, or soup [24], thereby indicating a lack of knowledge regarding the action of snake venom.

*Testudo graeca* Linnaeus, 1758 (Fig. 5d): The tortoise is the most commonly used reptile for medicinal and magical purposes, and the reptile most frequently used by herbalists in Moroccan medinas [10]. They are used for favoring the cessation of breast feeding in children. The belief in its utility for aiding delayed onset of walking in children and to remove curses relates to the tortoise’s ability to walk slowly but surely, and to the shell that protects them from danger (see also [43], use already pointed out by ancient physicians [58]). Moroccans also believe that the magic power of a tortoise surpasses that of a snake, which provides another reason to keep tortoises in one’s courtyard (to scare away snakes [77]). Because they are considered devils, the inhaled smoke from a burned tortoise carapace is believed to cure those afflicted by witchcraft [24, 43]. Dioscorides also cited that turtle’s blood is good for epileptics [65], while Al-Baytar mentioned uses for the treatment of whooping cough, skin ulcers, burns, epilepsy, asthma, convulsions, tetanus, cataracts, and hernias from previous authors [58]. In the Rif, tortoises are killed exclusively for medicinal or sorcery uses and are more commonly taken from the wild than purchased in souks. Tortoises in North Africa are believed to be coprophagous [77]. This dietary habit currently renders them impure in accordance with Muslim traditions, and suggests that ethnozoological use in the region precedes the arrival of Islam.

#### Aves

*Athene noctua* (Scopoli, 1769): Its eyes may be used to combat insomnia, and depending upon whether the right eye or the left eye is involved, insomnia or sleep may be affected (see also [43]). When present elsewhere within Morocco, the species is reputed to attract natural disasters [43].

*Alectoris barbara* (Bonnaterre, 1792): Its gall is used topically to treat watery eyes in other areas of Morocco [43]. Partridges are mentioned in works by both Dioscorides and Al-Baytar, first in chapters devoted to the blood and bile, specifying that “the blood of [several species of birds], when fresh, are anointed in eye injuries, bleedings, and to improve poor night vision”, and the bile is good for “cataracts, opacities, and white spots on the cornea” [65]. Nevertheless, partridges in Al-Baytar’s work [58] are mentioned several times without any coincidences of uses.

*Bubulcus ibis* (Linnaeus, 1758): Its use does not appear in other ethnozoological studies of Morocco.

*Columba livia* Gmelin, 1789: In Fez, the Beni Sadden cook it without salt to treat diseases caused by demons [43]. Al-Baytar cited that, according to previous authors [58], it is used for the treatment of the kidney, blood, sperm, eye, and skin conditions (including burns, pimples, to mitigate scars, and scorpion bites); ringworm; dysuria; and calculi, apart from its mentions as a good source of food.

*Corvus corax* Linnaeus, 1758: The bird’s head hanging on the door of a patient accelerates healing, but burying the animal in front of an enemy’s house attracts discomfort, and the sight of the bird during a trip brings unhappiness [24]. In Morocco, there is a belief that in order to achieve the desired curative and/or magical effect, the bird must be killed without the animal seeing the hunter. If the hunter is seen, a “sorcery substance” that serves as medicine will not be available because the crow drinks its own gall, the source of a substance acting as an antipyretic, and the “sorcery substance” is no longer produced when the bird is dying from a gunshot wound [43].

*Gallus gallus* (Linnaeus, 1758): In other parts of Morocco, its egg shell is used for sorcery and the ingestion of its liver causes cowardice [43]. Chicken soup is considered a general curative, which was also indicated by several ancient physicians like Galen, Razes, Dioscorides, and Al-Baytar [58], among other medicinal properties of the animal.

*Upupa epops* Linnaeus, 1758: Hoopoe had the most ethnozoological uses of any bird in this region (Table 1). In general, Eurasian hoopoe has several important sorcery and medicinal uses in Morocco. For example, eating the heart strengthens memory [23, 43], and a dried head or body hung from the neck protects the wearer from the evil eye and misfortune (such as theft) while increasing good luck [23, 24, 43]. *U. epops’* song “hut hut hut” emulates an expression from the Berber language meaning “there, there, there,” and from this song, the supposed ability to see buried treasures has arisen [43]. Eyes are used in rituals involved in avoidance of the evil eye [23]. Among the Ait Ouarin, a person wearing the dry head may either be seen by others as more pleasant or may cause others to fear them, and the dry head generally prevents other sorcery products, such as milk or butter, from being cursed by opponents [43]. *U. epops* is sold in some souks in the Rif at high prices (2000 dh [Moroccan currency]) because of its many medicinal and sorcery uses. The relation to sorcery and the attraction of good luck must be very old, as the use of its eyes was mentioned by Ibn Zuhr (or Avenzoar; Andalusian physician, 1094–1162, cited by Al-Baytar [58]) to recover memory, treat leprosy, and attract victory in conflicts magically, as well as other parts or the whole animal to attract good luck or avoid the evil eye in houses.

#### Mammalia

*Atelerix algirus* Lereboullet, 1842: The hedgehog is the third most cited species among Riffians (Table 1), and the only species with sorcery, medicinal, and veterinary uses that has been cited throughout most of Morocco. Body spikes are ritualistically burned to repel the evil eye (possibly related to its “rolling into a ball” defense mechanism). Meat consumed by sick people has an invigorating effect [24], particularly in the Yebala region [78]. The blood heals ringworm, warts, and cracked skin on the feet [23, 43]. When consumed by young people, hair growth is favored as if it were hedgehog spikes. When burned, ashes sifted, mixed with henna, and applied to women’s hair help to strengthen it [43]. In contrast to other regions of Morocco, hunting for hedgehogs in the Rif is more popular than buying them [26]. For Riffians, the cunning of the hedgehog is superior to that of the wolf [63]. Some classical authors described uses for the hedgehog: Dioscorides mentioned it for the treatment of alopecia [65], and Ibn Sina cited it (c. 980-1037) for the treatment of scrofula [58], but no uses relating to rituals or sorcery were mentioned. Thus, we suppose this relation is specific to the Moroccan culture.

*Bos taurus* Linnaeus, 1758: Cattle represent one species for which utilization differs by sex (Table 1). Authors in antiquity highlighted its nutritional properties, as one of the best meats, and as invigorative [58], and mentioned several uses for its meat and milk, and all of them coincide with our findings in the studied area.

*Camelus dromedarius* Linnaeus, 1758 (Fig. 5f): Camels are the mammalian species with the most ethnozoological uses in this region. The wide variety of medicinal uses among Riffians does not match the ways in which the hump fat is utilized by other Berbers of northwestern Africa, the Sahrawis of Western Sahara (otitis, earache, and open wounds [25];). The gates of Tangier’s city walls were covered in camel skin during the nineteenth century, and inhabitants cut pieces from the skins for use in sorcery and medicinal rites [18]. In other areas of Morocco, it is believed that hiccups and whooping cough can be prevented and cured by hanging a small piece of trachea from a child’s neck or drinking camel’s blood [23, 43]. Consuming camel brains supposedly cures ringworm, drinking urine may cure fevers, pulverized bones are used for scabies, and camel meat can be used to heal boils and can strengthen the body in general, since it is considered a strong animal. Children who fear physical punishment by teachers in the madrasah seek out camels to have them urinate on their legs so as to render them insensitive to pain. Additionally, after placing a stick in its mouth to prevent it from biting, putting one’s hands inside a camel’s mouth will eliminate the evil eye [43]. Hiding camel flesh in a patient’s food ensures his infatuation [23], and drinking camel’s milk flavored with sesame will help thin women to gain weight [24]. As for the cow and bull, Al-Baytar compiled the uses for these animals which most important physicians from antiquity later mentioned, without any overlap with the uses in our study area. Its fat was also mentioned by Dioscorides [65]. Contrary to our findings, its meat was considered to weaken eyesight in the past [58].

*Canis lupaster* Hemprich & Ehrenberg, 1833 (Fig. 5g): All of the uses for the African wolf could be derived from beliefs associated with its thick coat of hair (see [43]). Wolf hide was frequently sold in souks of the Rif at the beginning of the twentieth century [22], though this no longer occurs. Consumption of the flesh of this species is prohibited by Islam, but it does happen, as noted by Drummond-Hay and Cabrera [18, 22], suggesting that these practices are ancient. In this region, African wolves were favored during the Neolithic expansion of the human population, and ethnozoologic utilization probably began well before the Arab conquest and the expansion of Islam [67]. In the Rif and throughout Morocco, however, wolves have undergone a population decrease in recent years, not due to ethnozoological uses but strychnine poisoning campaigns [79]. There are no coincident uses with those compiled by Ibn al-Baytar.

*Canis lupus familiaris* Linnaeus, 1758: There are many additional ethnozoological uses for this species found elsewhere in Morocco, and Westermarck [43] cites 52 properties, half of them considered kind and half of them considered evil. Women, for example, ingest the meat of male puppies as a cure for infertility, or drink an adult male dog’s urine to ensure the birth of a baby. The superstitious anti-poison and health-improving virtues of dogs were also recorded by the Andalusian Ibn Zhur (Avenzoar, c.1073-c.1161). Teeth can be used to heal people who talk in their sleep, and a dog-tooth carried by a child makes their milk teeth fall out. Dog’s teeth are useful for treating jaundice, all kinds of accidents, and especially rabies [58]. According to Islamic law, dogs are impure animals that should not be consumed, so these actions must be pre-Islamic.

*Capra aegagrus* Erxleben, 1777: The ingestion of goat meat is considered an antidote for poisoning (see also [43]). Children of the Anjra tribe, adjacent to the study area, were forbidden to eat goat hearts because they cause permanent pimples. However, eating goat liver cures night blindness [43] (goats are considered to possess good night vision, a use described in Dioscorides [65]), and after sacrificing a goat, residual manure from its intestines serves as an antidote for spider bites, as this manure is considered to be a purifier [43]. Sahrawis from Western Sahara use its milk to treat otitis, earache, and nosebleeds [25]. Riffians consider goats to be evil, and incantations to induce nightmares were written on paper using the blood of a black male goat [43]. Fear reduction may be accomplished by burning goat droppings, seven stones, and seven units of coal in a container placed on top of the affected person. Disembodied spirits manifest themselves in the form of this animal, and the female mythological figure *Aicha Kandicha* is envisioned as a beautiful woman with goat’s feet.

*Equus asinus* Linnaeus, 1758: Donkey ears are used to stupefy and make people obedient. For example, a violent or inattentive husband will become obedient like a donkey after eating donkey ears (see also [43]).

*Equus caballus* Linnaeus, 1758: In general, the horse is considered a saint and family member, not simply a work animal [18]. In Fez, young women rubbed their vulvas with horse saliva to prevent pubic hair growth, and in Tangier, face washing with water which was just used by a horse prevented eye disease [43].

*Felis lybica* Forster, 1780: People from the Anjra tribe (Western Rif) believed that eating a cat’s leftover food was good for nervousness, and in Tangier, it was believed that to wash with a water source that a wildcat had just drank from was healthy. Among the Beni Waryager, cat meat was consumed as a general curative, and among the Temsaman, the freshly cut stomach of a cat served as protection from poisoning [43]. Throughout Morocco, black cats’ blood was used for writing spells, and its meat was served to prisoners so that they could be released [43].

*Gazella cuvieri* Ogilvy, 1840: Gazelle meat was served as a diuretic, a use not currently practiced due to its regional extinction [35]. South of the Rif, among the Ait Ouarain, when a gazelle was caught, pregnant women were taken to stare into its eyes for a long time, which favored having a baby with large black eyes like the animal’s [43]. In Marrakech, pieces of gazelle skin upon which to write spells that prevent evil eyes or disease were sold (and this continues today; author’s per. obs.) [23].

*Hyaena hyaena* Linnaeus, 1758: Moroccans considered the hyena cowardly and stupid [18, 22] and, as with donkey ears, its ears were used to stupefy the consumer [43]. Other parts of the animal were used for sorcery in Morocco [64]. There is also the belief that hyenas have magical powers, such as hypnotizing and devouring those who make eye contact with it. Depending upon the tribe involved, there are different ways to fall prey to its magic: in Ait Ouarain, when urine splashed by its tail is touched, and in Beni Waryager, when it makes noise while one is hunting it [22]. In Temsaman, because dogs are frightened when perceiving hyena odors, thieves used hyena fur to avoid guard dogs [22]. Products made from hyena are very expensive in Moroccan herbal markets, because of their current shortage in the country [35].

*Mustela nivalis* Linnaeus, 1766: Dried skins and bodies were sold in souks for veterinary and medicinal use elsewhere in Morocco [23, 43]. Weasels were also mentioned by Dioscorides and Al-Baytar [58, 65] as anti-venom.

*Oryctolagus cuniculus* Linnaeus, 1758: In Beni Waryager, the liver was roasted and cut into three parts by the village teacher, who wrote Quran prayers on them. The patient then had to eat one prayer every day, and in the evening, share it with his dog to cure himself of night blindness [43].

*Ovis aries* Linnaeus, 1758: In Fez, ram horns were hung from pomegranate trees (*Punica granatum* L.) to prevent the fruits from falling [43].

*Panthera leo* Linnaeus, 1758: Riffians consider the lion to be a noble and clever animal [18] and ate its meat to gain courage [43]. The lion was abundant in many regions of Morocco until the nineteenth century [18, 80], but disappeared from North Africa during the mid-twentieth century [81]. Our record from the Eastern Rif indicates the survival of these ethnozoological uses in Berber memory.

*Sus scrofa* Linnaeus, 1758: Because they have the ability to eat everything, this species’ intestines are used. Tusks serve as amulets because “evil eyes will focus on the tusk and not on the child” (see also [18]). As it is considered a very strong animal, consumption of its meat strengthens people (see also [78]). In fact, strong or warrior-like people were flattered by being called *jaluf* (wild boar). In other regions of Morocco, the Ait Ouarain and the Glaoua used their meat as a tonic for weak children. The Glaoua believed they acquired immunity to pain and punishment by ingesting hog meat [43]. In Fez, for veterinary use, horses were supplied with boar meat to gain strength [43], and in general, dogs were also fed with the meat [18]. Despite being an animal whose consumption is prohibited for Muslims, Berbers do not believe they are breaking the rule when they hunt and consume boars, and this activity is currently practiced clandestinely [78]. Wild boar recipes are probably very old, dating from before the arrival of Arabs to the region.

*Vulpes vulpes* Linnaeus, 1758 (Fig. 5h): Ingestion of cooked red fox meat, sifting their ashes and mixing them with honey, or drinking their blood serves as treatment for asthma (see also [64]). In the rest of Morocco, red foxes had numerous ethnozoological uses [43]. Among the Ait Ouarain, its fat was applied topically in the ears of the deaf to enable hearing (“due to the excellent hearing” of foxes; as described by Dioscorides, [65]), on the extremities to prevent hair growth, and on the chest and pubis to prevent diseases related to these areas. Cooked brain and ashes were ingested to treat syphilis. The use of its meat to treat joint pains was already described by Ibn Sina (cited in Al-Baytar [58]). In the Rif, it is not frequently sold in souks, as it is trapped by hunters. In the rest of Morocco, however, its skins are the most frequently sold in the medinas and souks of any carnivore [27].

### Animal body parts and modes of application

The use of 21 parts of animals’ bodies have been recorded (*n* = 193; Table 1), though utilization of the entire body, meat, or blood predominates (Figure S1). Eighteen modes of application of animal products were recorded (*n* = 190, Table 1). Ingestion in the form of stew predominates (suggesting that they are functional foods), followed by drinking, ingestion of ashes, and topical application (Figure S2). Although ophidian venom is ineffective as a killing agent after it has been heated or subjected to digestive enzymes, venom was supplied as an agent to be used for murdering an enemy.

### Diseases treated by ethnozoology and magico-religious medicinal uses

**Fig. 6:**
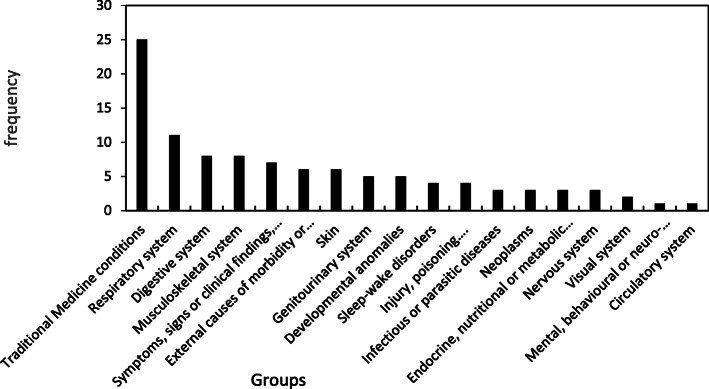
Pathological groups for medicinal uses (*n* = 105). Conditions grouped according the WHO International Classification of Diseases (ICD-11 version; Table S1)

Among the described medicinal uses, treatments have been found for 18 groups of human diseases (*n* = 105) out of the 26 in the classification used (Fig. 6; Table S1). Diseases related to the traditional medicinal system were most frequently cited. This includes uses for “general healing” (with ten animals) and “snake repellent,” as well as those described as treatment to dispel the “evil eye,” “fear,” and “bad luck,” as “sorcery,” or to “recover after fall” (25 total uses). These last examples can clearly be considered culture-bound syndromes related to local folklore (see [41, 60, 82] for theoretical considerations).

Conditions related to the respiratory system reached the second position, followed by those related to the digestive system, musculoskeletal system, and the category for symptoms, signs, or clinical findings not elsewhere classified (like fever). Most diseases of the respiratory system are related to asthma, probably because some houses in rural areas have wooden stoves that cause pulmonary irritation (see [51]). In regards to ailments involving the endocrine system, cessation of lactation by children is sought (which promotes more frequent procreation), and development of diabetes is frequent among elderly Riffians [73]. Among ailments related to the digestive system, diarrhea and stomach and intestinal pain are likely related to poor hygiene when handling food. Additionally, judging from the high number of medicinal plants used by healers, these problems must be fairly common [12, 45, 74, 83]. Ethnozoological uses affecting mental or behavioral diseases are those intended to stupefy others, or those granting historical recourse for women against their husbands. Among nomadic Berbers, however, nervous troubles are treated traditionally by ethnopharmacology [83]. Lice are the most frequently cited parasites, a consequence of the coexistence of some groups with livestock. Assistance with sleep or waking is generally meant to promote more effective nighttime livestock monitoring, which is especially important for those tribes partially dedicated to extensive livestock farming in a country where livestock predators have, until recently, maintained healthy populations [18, 35, 67]. Treatments for the eyes are meant to improve eyesight while hunting [78]. Among skin diseases, leukoderma is mentioned, and treatments for rheumatism are noted in relation to diseases of the musculoskeletal system. Urogenital problems involving the prostate and kidneys appear in older men. Due to the abundance of the Moorish viper, anti-venom treatments are common [32]. Unlike in other studies regarding ethnozoology in Morocco [18, 21, 23, 24, 64, 78], we obtained no information on the treatment of problems involving hematology, immunology, or childbirth.

Regardless of the cultural diseases or culture-bound syndromes mentioned and how local inhabitants treat them, 32 (of 107) uses for treatment of any condition from any of the use categories are considered magico-religious (“M” in table 1). Among them, 18 are not used to treat a culture-bound syndrome, but a regular medical condition. These are cases in which the healing process cannot be explained on the basis of alleged pharmacological activity (i.e., the ingestion of an animal part containing specific chemical compounds with specific pharmacological activities). Some of the uses could be related to the Doctrine of Signatures, a well-known topic regarding plant use [84] but scarcely considered in regards to animal use, and even then not on the basis of the animal’s shape but on the function of certain organs. For example, hedgehog spines (the main protection for the animal) were used to protect against the evil eye. Nevertheless, we consider these relationships by focusing more on the basis of the “similia similibus curantur” theory. Examples include the elimination of eye tearing by use of a snake’s shed skin (snakes are believed to have “clean eyes”), burning hedgehog spikes to strengthen human hair, eating camel meat to gain strength (like a camel), consuming boar intestines to combat intestinal pain, washing a baby over a tortoise to avoid walking delay (as tortoises walk slowly but surely), eating raven meat to improve sight (due to the animal’s reputation for good vision), and the meat of an African wolf used to stay awake, because the animal is active at all hours of the night (see chapter 3.2).

### Current extent and historical analysis of uses

It is noteworthy that 76% of uses were described as not currently practiced by at least one informant (Table 1). Thus, most of the uses described remain in the culture as ancient folk traditions. There may be different reasons for the abandonment of these uses, such as the recent introduction of allopathic medicine ([11]; per. obs. of authors). Respondents reporting on now-discontinued practices currently believe in modern medicine and tend to discard ethnozoological practices. Some of our respondents commented that if we had asked only ten years earlier, they would have mentioned more recipes and more uses, due to elderly holders of that knowledge having died during this time. Recording the cultural heritage of isolated populations is therefore fully justified; the longer we wait, the more traditional and historical information we lose [70, 85]. In our opinion, this knowledge should be documented and preserved as part of the intangible heritage of the Berbers, even if the practices are lost through cessation. We have been consistently and warmly received by survey respondents, a fact that we interpret as an acknowledgment of the importance the natives hold with regard to this facet of their culture.

In the literature analysis, we found citations for most of the animals included in the current study in the works of Dioscorides and Ibn al-Baytar, but with few coinciding uses. While the *Materia Medica* cites 61 animals from which medicinal remedies can be extracted [86] (22 of them mentioned by our informants), Ibn al-Baytar’s compendium, including previous annotations from at least 41 authors from Aristoteles and Dioscorides to Razes and Ibn Sina [87], cites up to 151 animals (24 of them mentioned by our informants, see table S3). Nevertheless, just a few uses coincide with those detailed in these sources, considering the part of the animal used and the mode of administration, as these sources are not always clear in those aspects. Three uses coincide with the texts by Dioscorides and Ibn-al Baytar, and three more only with the second one (Table 1). These observations, along with the high proportion of magico-religious uses (Section 3.4), suggest that traditional medicine in the Eastern Rif is not based upon the Hippocratic-Galenic procedures adopted and followed by Muslins in the Middle Ages [70], and reinforces the idea that Riffian medical traditions are ancient, and have developed in isolation [21].

In an effort to relate our findings to the current religious context of the study area, we looked for the animals mentioned in the Quran. The Holy Book mentions 31 animals [88], of which 16 were also cited in this research (see table S3): the bee, bird, calf and cow, camel (cited in up to 15 times), raven, dog, donkey, frog, goat, hoopoe, horse, lion, pig, sheep, snake, and wolf. Nonetheless, we have highlighted some uses described that in some way do not follow the religious precepts of Islam throughout the text (e.g., eating meats from animals considered impure like dogs, wolves, wild boars, and hyenas, as well as the consumption of the blood and urine of some animals). This finding could be seen as ancient reminiscences of Berber paganism with respect to Islam.

### Origin of utilized animals and implications for regional biodiversity

Wild animals required for recipes are mostly obtained by hunting (*A. algirus, A. barbara*, *A. mauritanicus*, *A. noctua, B. ibis, C. chameleon, C. corax, C. livia, C. lupaster, G. cuvieri, H. hyena, M. nivalis, O. cuniculus, P. algirus, P. leo,* ophidians*, S. scrofa, T. graea, U. epops, V. vulpes*), though animals extinct in the region (*G. cuvieri*, *H. hyaena*, *P. leo)* are obtained in souks [35]. In the past, when currently locally extinct animal species thrived in the region, all species used by Riffians were of local origin as a consequence of isolation within the mountainous region of the Eastern Rif and were obtained using traditional hunting skills [89]. Ethnozoological studies on tribal and other isolated communities typically found most utilized species to be wild caught (e.g., in Brazil [90]), while studies in Mediterranean regions located along secular trading routes found that many imported products were included (e.g., in Jordan [70]). There is information on ethnozoological products sold in Moroccan stores, but this does not cover areas inhabited by Berbers [27, 28, 64, 77, 91].

One of 20 wild species extant in the Rif and used in ethnozoology, *T. graeca*, is globally threatened (Vulnerable, IUCN Red List category [92];), although considered Low Concern at the regional level of Morocco [57]. Two more species utilized are globally threatened (*P. leo* and *G. cuvieri,* both vulnerable), but are regionally extinct. They disappeared from the region because of hunting, not due to exploitation for ethnozoology [35]. The impact of Riffian ethnozoology on both species would be through trade of their body parts obtained from populations outside of the Rif Mountains. Most of the citations regarding the conservation status of the species from the interviews (78.7%; *n* = 150) indicate that those used in regional ethnozoology are not in decline. *Vulpes vulpes* and *C. lupaster* are exceptions, not because of their importance to ethnozoology, but rather because they prey upon livestock, and are the victims of poisoning campaigns [35, 79]. Thus, potential damage to regional biodiversity resulting from contemporary ethnozoological practices seems to be low (see [5]). Moreover, we did not record any animal deaths in the region resulting from poor conditions in captivity, something that cannot be said for herbalists in the medinas of Western Morocco [28].

## Conclusions

Ethnic Berbers in the mountainous Eastern Rif maintain a rather rich culture of ethnozoological practices based on terrestrial vertebrates, mostly mammals and birds with a lesser presence of reptiles, amphibians, and invertebrates. We failed to record the use of continental fishes.

Ethnozoological use consisting of ingestion of some parts of animals (e.g., stewed meat) to facilitate a positive therapeutic effect for sick and debilitated people was expected. Most uses, however, are pure folk beliefs based on the supposedly magical/sorcery properties of animals [24, 43], some derived from similarities between human and animal morphology and behavior and the illness or ailment being treated. Their therapeutic effect is mainly confined to a placebo effect [41]. Some of the animals included were already mentioned as medicinal in Dioscorides and Ibn al-Baytar’s medicinal works, although these practices mostly do not coincide with our findings. Moreover, ailments treated and animal parts used by Riffians scarcely coincide with those used by people of Arabic origin within the Mediterranean Basin [16, 45, 70].

Perhaps the most interesting of our findings is that Berbers of the Eastern Rif preserve ethnozoological uses anathema to Islam, uses that continue in spite of the arrival of Arabs, unlike forbidden ethnobotanical uses in the Atlantic plains of Western Morocco [12]. Among these forbidden uses, consumption of impure animals (dog blood and urine, meat of dogs, wolves, wild boars, and hyenas) is included. The arrival and conquest of the region by Arabs occurred at the end of the seventh century, when all Maghrebians were Islamized. Berber paganism (with respect to Islam), however, was apparently not completely eliminated [21], and some ancestral habits have been maintained over time without change after Islamization by the Arabs, as demonstrated by our study of ethnozoology in the region. We advocate for other ethnozoological studies in the remaining mountain ranges of Morocco (Middle Atlas, High Atlas, Anti Atlas), also inhabited by Berbers, before this knowledge vanishes.

## Supplementary Information

**Additional file 1: Table S1.** International Classification of Diseases by the World Health Organization (ICD-11 version; OMS 2019). Codes as in Table 1 in the main text. **Table S2.** Species of amphibians, reptiles, birds, and mammals (only wild species) extant or recently extinct on the Eastern Rif (N Morocco). Phenology included for birds. Species are arranged in alphabetical order within taxonomic groups, for the readers’ convenience. See Materials and Methods section for the sources. **Figure S1.** Animal parts used. Numbers above bars represent percentages (*n* = 193). **Figure S2.** Application modes. Numbers above bars represent percentages (*n* = 190). **Table S3.** Animals from this study also mentioned in the consulted historical sources: Dioscorides, Ibn al-Baytar, and The Quran (see text for references and details). For unclear associations with the species, we provide the vernacular name from the original source in the list in brackets.

## Data Availability

All data used to support our findings are presented in this paper.
