# Supplementary material for: Ethnozoology among the Berbers: pre-Islamic practices survive in the Rif (northwestern Africa)
Source: J Ethnobiol Ethnomed. 2021 Jul 13;17:43. doi: 10.1186/s13002-021-00466-9 (PMC8278736; doi:10.1186/s13002-021-00466-9)
Supplement: Supplementary file 1 — Additional file 1: Table S1. International Classification of Diseases by the World Health Organization (ICD-11 version; OMS 2019). Codes as in Table 1 in the main text. Table S2. Species of amphibians, reptiles, birds, and mammals (only wild species) extant or recently extinct on the Eastern Rif (N Morocco). Phenology included for birds. Species are arranged in alphabetical order within taxonomic groups, for the readers’ convenience. See Materials and Methods section for the sources. Figure S1. Animal parts used. Numbers above bars represent percentages (n = 193). Figure S2. Application modes. Numbers above bars represent percentages (n = 190). Table S3. Animals from this study also mentioned in the consulted historical sources: Dioscorides, Ibn al-Baytar, and The Quran (see text for references and details). For unclear associations with the species, we provide the vernacular name from the original source in the list in brackets. [file 13002_2021_466_MOESM1_ESM.docx]

**Ethnozoology among the Berbers: pre-Islamic practices survive in the Rif (northwestern Africa)**

Aymane Budjaj^1^, Guillermo Benítez^*2^, Juan Manuel Pleguezuelos^1^

^1^ Dep. of Zoology, Fac. of Sciences, University of Granada, 18071 Granada, Spain

^2^ Dep. of Botany, Fac. of Pharmacy, University of Granada, 18071 Granada, Spain

*Corresponding author.

E-mail addresses: [aymane.boudjajjovellanos@gmail.com](mailto:aymane.boudjajjovellanos@gmail.com) (A. Budjaj), [gbcruz@ugr.es](mailto:gbcruz@ugr.es) (G. Benítez), [juanple@ugr.es](mailto:juanple@ugr.es) (J.M. Pleguezuelos).

Supplementary Material

Table S1. International Classification of Diseases by the World Health Organization (ICD-11 version; OMS 2019). Codes as in Table 1 in the main text.

Codes Diseases

1 Certain infectious or parasitic diseases

2 Neoplasms

3 Diseases of the blood or blood-forming organs

4 Diseases of the immune system

5 Endocrine, nutritional or metabolic diseases

6 Mental, behavioural or neuro-developmental disorders

7 Sleep-wake disorders

8 Diseases of the nervous system

9 Diseases of the visual system

10 Diseases of the ear or mastoid process

11 Diseases of the circulatory system

12 Diseases of the respiratory system

13 Diseases of the digestive system

14 Diseases of the skin

15 Diseases of the musculoskeletal system or connective tissue

16 Diseases of the genitourinary system

17 Conditions related to sexual health

18 Pregnancy, childbirth or the puerperium

19 Certain conditions originating in the perinatal period

20 Developmental anomalies

21 Symptoms, signs or clinical findings, not elsewhere classified

22 Injury, poisoning or certain other consequences of external causes

23 External causes of morbidity or mortality

24 Factors influencing health status or contact with health services

25 Codes for special purposes

26 Traditional Medicine conditions

Table S2. Species of amphibians, reptiles, birds, and mammals (only wild species) extant or recently extinct on the Eastern Rif (N Morocco). Phenology included for birds. Species are arranged in alphabetical order within taxonomic groups, for the readers’ convenience. See Materials and Methods section for the sources.

Scientific name Common name

Amphibians

1. *Alytes maurus*  Moroccan midwife toad
2. *Bufotes boulengeri* African green toad
3. *Pelophylax saharicus* North African frog
4. *Sclerophrys mauritanica* Moroccan toad

Reptiles

1. *Acanthodactylus erythryrus* Fringe-fingered lizard
2. *Agama impalearis* Bibron’s agama
3. *Chalcides colosii*  Colosi’s skink
4. *Chalcides ocellatus* Ocellated skink
5. *Chamaeleo chamaeleon* Common chameleon
6. *Coronella girondica* Southern smooth snake
7. *Daboia mauritanica*  Moorish viper
8. *Eumeces algeriensis*  Algerian skink
9. *Hemidactylus turcicus* Mediterranean house gecko
10. *Hemorrhois hippocrepis* Horseshoe whip snake
11. *Macroprotodon brevis*  Western false smoth snake
12. *Malpolon monspessulanus* Montpellier snake
13. *Mauremys leprosa*  Mediterranean turtle
14. *Natrix maura*  Viperine snake
15. *Podarcis vaucheri* Andalusian Wall lizard
16. *Psammodromus algirus*  Algerian psammodromus
17. *Psammodromus blanci*  Blank’s psammodromus
18. *Psammophis schokari*  Shokari sand racer
19. *Saurodactylus mauritanicus* Morocco lizard-fingered Gecko
20. *Tarentola mauritanica*  Moorish gecko
21. *Testudo graeca*  Mediterranean spur-thighed tortoise
22. *Timon tangitanus* Moroccan eyed lizard
23. *Trogonopshis wiegmanni* Checkedboard worm lizard
24. *Uromastyx nigriventris* Moroccan spiny-tail lizard

Aves

1. *Accipiter nisus* Eurasian sparrow hawk sedentary
2. *Actitis hypoleucos* Common sandpiper winter visitor
3. *Alauda arvensis* Eurasian skylark sedentary
4. *Alaudala rufescens* Lesser short-toed lark sedentary
5. *Alectoris barbara* Barbary partridge sedentary
6. *Anas clypeata* Northern shoveler winter visitor
7. *Anas platyrhynchos* Mallard winter visitor
8. *Anthus campestris* Tawny pipit summer visitor
9. *Anthus pratensis* Meadow pipit winter visitor
10. *Apus affinis* White-rumped swift sedentary
11. *Apus apus* Little swift summer visitor
12. *Apus pallidus* Pallid swift summer visitor
13. *Aquila chrysaetos* Golden eagle sedentary
14. *Aquila fasciata* Bonelli's eagle sedentary
15. *Ardea cinerea* Grey heron winter visitor
16. *Asio otus* Long-eared owl sedentary
17. *Athene noctua* Little owl sedentary
18. *Aythya ferina* Common pochard winter visitor
19. *Bubo bubo* Eurasian eagle-owl sedentary
20. *Bubulcus ibis* Cattle egret sedentary
21. *Bucanetes githagineus* Trumpeter finch sedentary
22. *Burhinus oedicnemus* Eurasian stone-curlew sedentary
23. *Buteo rufinus* Long-legged buzzard sedentary
24. *Calandrella brachydactyla* Greater short-toed lark summer visitor
25. *Caprimulgus europaeus* European nightjar summer visitor
26. *Caprimulgus ruficollis* Red-necked nightjar summer visitor
27. *Carduelis carduelis* European goldfinch sedentary
28. *Cecropis daurica* Red-rumped swallow summer visitor
29. *Cercotrichas galactotes* Rufous-tailed Scrub Robin summer visitor
30. *Certhia brachydactyla* Short-toed tree creeper sedentary
31. *Cettia cetti* Cetti’s warbler sedentary
32. *Charadrius dubius* Little ringed plover summer visitor
33. *Chloris chloris* European greenfinch sedentary
34. *Ciconia ciconia* White stork summer visitor
35. *Circaetus gallicus* Short-toed snake eagle summer visitor
36. *Cisticola juncidis* Zitting’s cisticola sedentary
37. *Coccothraustes coccothraustes* Hawfinch sedentary
38. *Columba livia* Rock dove sedentary
39. *Columba palumbus* Common Wood pigeon sedentary
40. *Coracias garrulus* European roller summer visitor
41. *Corvus corax* Northern raven sedentary
42. *Corvus monedula* Western jackdaw sedentary
43. *Coturnix coturnix* Common quail sedentary
44. *Cuculus canorus* Common cuckoo summer visitor
45. *Cyanistes caeruleus* Eurasian blue tit sedentary
46. *Delichon urbica* Common house martin summer visitor
47. *Egretta garzetta* Little egret sedentary
48. *Emberiza cia* Rock bunting sedentary
49. *Emberiza cirlus* Cirl bunting sedentary
50. *Erithacus rubecula* European robin winter visitor
51. *Falco naumanni* Lesser kestrel summer visitor
52. *Falco peregrinus* Peregrine sedentary
53. *Falco subbuteo*  Eurasian hobby summer visitor
54. *Falco tinnunculus* Kestrel sedentary
55. *Fringilla coelebs* Common chaffinch sedentary
56. *Fulica atra* Eurasian coot winter visitor
57. *Galerida cristata* Crested lark sedentary
58. *Galerida theklae* Thekla lark sedentary
59. *Gallinago gallinago* Common snipe winter visitor
60. *Gallinula chloropus* Common moorhen sedentary
61. *Garrulus glandarius* Eurasian jay sedentary
62. *Hieraaetus pennatus* Booted eagle summer visitor
63. *Hippolais polyglotta* Melodious warbler summer visitor
64. *Hirundo rustica* Barn swallow summer visitor
65. *Iduna pallida* Eastern olivaceous warbler summer visitor
66. *Lanius excubitor* Great grey shrike sedentary
67. *Lanius senator* Woodchat shrike summer visitor
68. *Larus ridibundus* Black-headed gull winter visitor
69. *Linaria cannabina* Common linnet sedentary
70. *Loxia curvirostra* Red crossbill sedentary
71. *Lullula arborea* Woodlark sedentary
72. *Luscinia megarhynchos* Common nightingale summer visitor
73. *Melanocorypha calandra* Calandra lark sedentary
74. *Merops apiaster* European bee-eater summer visitor
75. *Miliaria calandra* Corn bunting sedentary
76. *Milvus migrans* Black kite summer visitor
77. *Monticola solitarius* Blue rock thrush sedentary
78. *Motacilla alba*  White wagtail winter visitor
79. *Motacilla cinerea* Grey wagtail sedentary
80. *Motacilla flava* Western yellow wagtail summer visitor
81. *Muscicapa striata* Spotted flycatcher summer visitor
82. *Oenanthe hispanica* Black-eared wheatear summer visitor
83. *Oenanthe leucura* Black wheatear sedentary
84. *Oriolus oriolus* Eurasian golden oriole summer visitor
85. *Otus scops* Eurasian scops owl summer visitor
86. *Pandion haliaetus* Osprey winter visitor, sedentar
87. *Parus major* Great tit sedentary
88. *Passer domesticus* Sparrow sedentary
89. *Passer hispaniolensis* Spanish sparrow sedentary
90. *Periparus ater* Coal tit sedentary
91. *Petronia petronia* Rock sparrow sedentary
92. *Phoenicurus moussieri* Moussier’s redstart sedentary
93. *Phoenicurus ochruros* Black redstart winter visitor
94. *Phylloscopus collybita* Common chiffchaff winter visitor
95. *Phylloscopus ibericus* Iberian chiffchaff sedentary
96. *Pica pica* Eurasian magpie sedentary
97. *Picus vaillantii* Leivaillant's green woodpecker sedentary
98. *Pterocles orientalis* Black-bellied sand grouse sedentary
99. *Ptyonoprogne rupestris* Eurasian crag martin sedentary
100. *Pycnonotus barbatus* Common bubul sedentary
101. *Pyrrhocorax pyrrhocorax* Red-billed chough sedentary
102. *Regulus ignicapillus* Common fire crest winter visitor
103. *Saxicola rubicola* European stonechat sedentary
104. *Scolopax rusticola* Eurasian woodcok winter visitor
105. *Serinus serinus* European serin sedentary
106. *Serinus spinus* Eurasian siskin winter visitor
107. *Streptopelia decaocto* Eurasian collared dove sedentary
108. *Streptopelia turtur* Eurasian turtle dove summer visitor
109. *Strix aluco* Tawny owl sedentary
110. *Sturnus unicolor* Spotedless starling sedentary
111. *Sturnus vulgaris* Common starling winter visitor
112. *Sylvia atricapilla* Eurasian blackcap winter visitor
113. *Sylvia cantillans* Subalpine warbler summer visitor
114. *Sylvia conspicillata* Spectacled warbler summer visitor
115. *Sylvia hortensis* Western orphean warbler summer visitor
116. *Sylvia melanocephala* Sardinian warbler sedentary
117. *Sylvia undata* Dartford warbler sedentary
118. *Tachybaptus ruficollis* Little grebe sedentary
119. *Tachymarptis melba* Alpine swift summer visitor
120. *Tchagra senegala* Black-crowned tchagra sedentary
121. *Tringa ochropus* Green sandpiper winter visitor
122. *Troglodytes troglodytes* Eurasian wren sedentary
123. *Turdus iliacus* Redwing winter visitor
124. *Turdus merula* Common blackbird sedentary
125. *Turdus philomelos* Song thrush winter visitor
126. *Turdus torquatus* Ring ouzel winter visitor
127. *Turdus viscivorus* Mistle thrush sedentary
128. *Tyto alba* Barn owl sedentary
129. Upupa epops Eurasian hoopoe sedentary
130. *Vanellus vanellus* Northern lapwing winter visitor

Mammals

1. *Apodemus sylvaticus* Wood mouse
2. *Atelerix algirus* Algerian hedgehog
3. *Canis lupaster* African wolf
4. *Caracal caracal* Caracal
5. *Crocidura russula* Greater white-toothed shrew
6. *Crocidura whitakeri* North African lesser white-toothed shrew
7. *Elephantulus rozeti* North African elephant-shrew
8. *Eliomys mumbyanus* Maghreb garden dormouse
9. *Eptesicus isabellinus* Isabelline serotine
10. *Felis lybica* African wild cat
11. *Gazella cuvieri* Edmi gazelle
12. *Genetta genetta* Genet
13. *Gerbillus campestris* North African jerbil
14. *Herpestes ichneumon* Egyptian mongoose
15. *Hyaena hyaena* Striped hyena
16. *Hypsugo savii* Savi's pipistrelle
17. *Hystrix cristata* Crested porcupine
18. *Jaculus orientalis* Greater Egyptian jerboa
19. *Lemniscomys barbarus* Barbary striped grass mouse
20. *Lepus mediterraneus* Maghreb hare
21. *Meriones shawii* Shaw's jird
22. *Miniopterus schreibersii* Schereiber's bat
23. *Mus musculus* House mouse
24. *Mus spretus* Algerian mouse
25. *Mustela nivalis* Weasel
26. *Myotis capaccinii* Long-fingered bat
27. *Myotis emarginatus* Geoffroy's bat
28. *Myotis nattereri*  Natterer’s bat
29. *Myotis punicus* Magrebian mouse-eared bat
30. *Oryctolagus cuniculus* Rabbit
31. *Panthera leo* Lion
32. *Pipistrellus kuhlii* Kuhl's pipistrelle
33. *Pipistrellus pipistrellus* Common pipistrelle
34. *Rattus norvegicus* Brown rat
35. *Rattus rattus* Black rat
36. *Rhinolophus euryale* Mediterranean horseshoe bat
37. *Rhinolophus ferrumequinum* Greater horseshoe bat
38. Rhinolophus hipposideros Lesser horseshoe bat
39. *Suncus etruscus* Pigmy white-toothed shrew
40. *Sus scrofa* Wild boar
41. *Tadarida teniotis* European free-tailed bat
42. *Vulpes vulpes* Common red fox

Figure S1 Animal parts used. Numbers above bars represent percentages (n = 193).

Figure S2 Application modes. Numbers above bars represent percentages (n = 190).

Table S3. Animals from this study also mentioned in the consulted historical sources: Dioscorides, Ibn al-Baytar, and The Quran (see text for references and details). For unclear associations with the species, we provide the vernacular name from the original source in the list in brackets.

| Scientific name (family) | Dioscorides  (22) | Ibn al-Baytar  (24) | Quran  (16) |
| --- | --- | --- | --- |
| **Insecta** | | | |
| *Apis mellifera* (Apidae) | X | X | X |
| Lampyridae |  |  |  |
| **Amphibia** | | | |
| *Sclerophrys mauritanica* (Bufonidae) | X (frog) | X (frog) | X (frog) |
| **Reptilia** | | | |
| *Chamaeleo chamaeleon* (Chamaeleonidae) | X | X |  |
| *Psammodromus algirus* (Lacertidae) | X (snake) | X (snake) |  |
| *Ophidia* |  |  | X |
| *Testudo graeca* (Testudinidae) | X | X |  |
| **Aves** | | | |
| *Athene noctua* (Strigidae) |  |  |  |
| *Alectoris barbara* (Phasianidae) | X | X |  |
| *Bubulcus ibis* (Ardeidae) |  |  |  |
| *Columba livia* (Columbinae) | X | X |  |
| *Corvus corax* (Corvidae) |  | X | X |
| *Gallus gallus* (Phasianidae) | X | X | X (bird) |
| *Upupa epops* (Upupidae) |  | X | X |
| **Mammalia** | | | |
| *Atelerix algirus* (Erinaceidae) | X | X |  |
| *Bos taurus* (Bovidae) | X | X | X |
| *Camelus dromedarius* (Camelidae) | X | X | X |
| *Canis lupaster* (Canidae) |  | X | X |
| *Canis lupus familiaris* (Canidae) | X | X | X |
| *Capra aegagrus* (Bovidae) | X | X | X |
| *Equus asinus* (Equidae) | X | X | X |
| *Equus caballus* (Equidae) | X | X | X |
| *Felis lybica* (Felidae) |  | X |  |
| *Gazella cuvieri* (Bovidae) | X | X |  |
| *Oryctolagus cuniculus* (Leporidae) |  |  |  |
| *Ovis aries* (Bovidae) | X | X | X |
| *Hyaena hyaena* (Hyaenidae) | X | X |  |
| *Mustela nivalis* (Mustelidae) | X | X |  |
| *Panthera leo* (Felidae) | X | X | X |
| *Sus scrofa* (Suidae) | X | X | X |
| *Vulpes vulpes* (Canidae) | X | X |  |
